# A pay-it-forward approach to improve feedback rate of HPV-based self-sampling in cervical cancer screening among women in ethnic minority regions of China: a randomized controlled trial protocol

**DOI:** 10.3389/fpsyt.2025.1586076

**Published:** 2025-05-29

**Authors:** Xueyuan Zhou, Rantong Bao, Xin Guan, Wenzhuo Li, Chenxin Yang, Ruijin Zhu, Hui Li, Xiaohua Wang

**Affiliations:** ^1^ Department of Genetics, Inner Mongolia Maternity and Child Health Care Hospital, Hohhot, China; ^2^ Center for Clinical Epidemiology Research, the Affiliated Hospital of Inner Mongolia Medical University, Hohhot, China; ^3^ School of Nursing, China Medical University, Shenyang, China; ^4^ Medical College, Qingdao University, Qingdao, China; ^5^ School of Health Management, China Medical University, Shenyang, China; ^6^ School of Nursing, Henan University of Science and Technology, Luoyang, China; ^7^ Nursing Department, Shandong Provincial Hospital Affiliated to Shandong First Medical University, Jinan, China

**Keywords:** HPV-based self-sampling, pay-it-forward, feedback rate, randomized controlled trail, ethnic minority regions

## Abstract

**Background:**

Human papillomavirus (HPV) infection is the primary cause of cervical cancer, and HPV self-sampling has emerged as a novel screening method with the potential to increase screening coverage and early detection of cervical cancer. However, the implementation of HPV self-sampling in China faces several challenges, including sociocultural factors, economic burdens, and low feedback rates. This study will aim to improve the feedback rate of HPV self-sampling results through a Pay-It-Forward approach and explore its impact on HPV positivity rates and subsequent treatment compliance.

**Methods:**

This study employs a randomized controlled trial (RCT) design, enrolling women aged 24 and older who have not received the HPV vaccine and have not undergone HPV self-sampling or cervical cancer screening in the past 12 months. Participants will be randomly divided into a control group (free distribution of HPV self-sampling kits) and an intervention group (pay up front for HPV self-sampling). The prepayment amount is 20 RMB, which is fully refunded upon completion of the self-sampling and feedback of results. The primary outcome measure is the feedback rate of HPV self-sampling results. The study is conducted at maternal and child health care and family planning service centers in Hohhot, Inner Mongolia Autonomous Region, with an expected enrollment of 108 participants.

**Discussion:**

The Pay-It-Forward approach is expected to significantly improve the feedback rate of HPV self-sampling results by enhancing participants’ psychological commitment and sense of responsibility. Additionally, this strategy may positively affect HPV positivity rates and subsequent treatment compliance. The innovation of this study lies in the first application of the Pay-It-Forward method to HPV self-sampling and the comprehensive analysis of urban-rural differences. The results of this study will provide a scientific basis for improving women’s cervical health awareness and optimizing HPV self-sampling intervention strategies, thereby promoting the widespread application of HPV self-sampling in cervical cancer screening.

**Trial registration:**

This study registered at ClinicalTrials.gov (ChiCTR2500095770) on January 13, 2025.

## 1 Global status of HPV infection

Human papillomavirus (HPV) is a common sexually transmitted virus. In 2007, an estimated 291 million women worldwide were infected with HPV (1, 2). The infection is particularly prevalent among sexually active women, especially those under 25, with the highest infection rate, which decreases with age (3). Studies show a positive correlation between HPV infection rates and the severity of cervical lesions (4). Additionally, HPV-related diseases impose substantial economic burdens on healthcare systems, with direct costs ranging from 47.16 million to 1.8 billion yuan (5).

In China, HPV epidemiology mirrors global trends but has unique aspects. Studies indicate an overall HPV infection rate of 13.1% - 18.8% in the general population, with high-risk HPV at 12.95% - 17.1% and low-risk HPV at about 3.28% (6). HPV is a major cause of cervical cancer (7).

## 2 The importance of HPV screening in cervical cancer prevention

Cervical cancer is preventable and controllable. In 2020, the World Health Assembly adopted the Global Strategy to Accelerate the Elimination of Cervical Cancer, setting the 90-70–90 goals for 2030: 90% of girls vaccinated against HPV by 15, 70% of women screened at least once by 35-45, and 90% of diagnosed women treated (8, 9). In China, these measures could prevent 7.509 million new cases and 2.529 million deaths between 2021 and 2100 (10).

Despite HPV vaccines’ effectiveness, regular screening remains vital. HPV screening detects infections and cervical intraepithelial neoplasia (CIN) early, enabling timely intervention (11). Regular screening significantly improves early cervical cancer detection and reduces advanced cancer incidence (12). Early identification of high-risk HPV infections allows for timely management, as these individuals are more likely to develop precancerous lesions or invasive cancer (13). Thus, regular HPV screening is key to reducing cervical cancer incidence and mortality (14).

## 3 Introduction and challenges of HPV-based self-sampling

HPV-Based Self-Sampling is an emerging method allowing women to collect samples at home for HPV testing, aiming to improve screening coverage in areas with limited resources or low compliance. It typically involves using specially designed tools to collect vaginal or cervical swabs, sent to labs for testing (15).

HPV-Based Self-Sampling offers several advantages. It provides privacy, allowing women to comfortably, reducing awkwardness and discomfort (16). Self-sampling is often painless and less expensive than traditional methods, increasing women’s willingness to participate (17). Studies show self-testing results are highly consistent with traditional clinical sampling, indicating it can effectively replace conventional screening, especially in enhancing coverage and early HPV detection (18).

However, challenges remain. The accuracy of self-sampling technology needs attention, as issues like insufficient sample collection or improper operation may affect results (19). Participant acceptance is also key; some women may be unfamiliar with or lack confidence in self-sampling methods, affecting participation rates (20). Ensuring timely sample submission and efficient lab processing during self-examination is crucial for result validity (21).

## 4 Current situation and challenges of HPV-based self-sampling in China

In China, HPV-Based Self-Sampling aims to improve screening coverage and early cervical cancer detection but faces multiple challenges. The Chinese government and health departments recognize the importance of HPV screening in cervical cancer prevention and have promoted this method. HPV testing is included in cervical cancer screening programs, with free or subsidized screening launched in some areas (22). These measures have improved women’s screening coverage, but the overall self-examination rate is still low, partly due to insufficient publicity, inconvenient transportation, and economic burdens (23). Despite subsidies, HPV-Based Self-Sampling’s nationwide coverage is far below that of many developed countries (24).

In Inner Mongolia, a region characterized by complex geography and a dispersed population, HPV-based self-sampling has shown significant potential in enhancing screening coverage. Studies have demonstrated that self-sampling is highly acceptable and effective in resource-limited settings, particularly in rural and remote areas where traditional screening methods face implementation challenges (25). This approach offers privacy, convenience, and cost-effectiveness, making it a promising strategy to overcome geographical and resource barriers in cervical cancer screening (25).

## 5 Factors affecting the feedback rate of HPV-based self-sampling results

Unique socio-cultural factors in China significantly influence the acceptance and feedback rate of HPV-Based Self-Sampling. Traditional beliefs and attitudes towards sexual health can create cognitive barriers and lead to low acceptance among women (26). Some women lack confidence in self-examination or awareness of HPV risks, affecting their compliance (27). Insufficient resource allocation in rural areas is also a major challenge, reducing rural women’s opportunities for HPV screening and subsequent treatment (28). Differences in HPV testing quality between regions and institutions, and a lack of unified quality control standards, may affect test accuracy and reliability (29). Socio-economic factors, such as income level and occupation type, significantly impact women’s willingness and ability to access and provide feedback on HPV screening services (30). Cultural and educational factors also play a role, in conservative cultures, women may feel ashamed about self-examination involving private parts, and less educated women may lack relevant health knowledge, leading to lower participation (26, 31). Healthcare system factors, including uneven medical resource distribution and varying screening service quality, further constrain women’s access to HPV screening services (32, 33).

## 6 Current intervention measures and limitations for improving the self-sampling feedback rate of female HPV

The refundable deposit mechanism, grounded in behavioral science and behavioral economics, leverages the sunk cost effect and present bias to enhance participant engagement (34). By requiring participants to pre-pay a fee that is refunded upon completing self-sampling and feedback, this strategy increases psychological commitment and motivation to complete the process. This approach not only reduces economic barriers but also promotes timely sample submission and feedback, thereby improving screening outcomes and participant compliance. Studies have shown that such mechanisms can significantly enhance adherence to health screening programs by creating a sense of responsibility and investment among participants (35).

Health education and publicity interventions, based on the Health Belief Model and Social Cognitive Theory, aim to improve the self-sampling feedback rate of female HPV. These interventions have been shown to increase self-sampling rates, particularly among low-income women through community health education programs and among young women via social media (36). However, their effectiveness is limited by information dissemination channels and audience health awareness levels (37), and their impact is often short-term, lacking mechanisms for long-term tracking and sustained intervention (38). Economic incentive measures, such as providing free self-sampling reagents or cash rewards, have significant short-term effects based on behavioral economics theory (39). These measures increase the feedback rate of self-sampling by reducing participants’ economic burden (38), but their long-term effects and fairness still need further exploration (40). Interventions to improve self-sampling reagents and techniques, based on the Technology Acceptance Model and User Experience Theory, aim to enhance user experience. However, their promotion and popularization still require time and resources, potentially leading to uneven coverage among different populations (37). Community support and mobilization, based on Social Capital Theory and Community Mobilization Theory, have played an important role in improving the feedback rate of HPV self-sampling. However, the effectiveness of this intervention depends on the adequacy of community resources and the ability to mobilize personnel, and in communities with limited resources, the implementation effect may be constrained (41).

However, the prepaid strategy, based on the Sunk Cost Effect and Present Bias, emphasizes the impact of economic input on behavioral persistence (42). Prepaid interventions have been shown to significantly improve the feedback rate of HPV self-sampling (37). This strategy enhances participants’ psychological commitment to self-sampling behavior and increases their motivation to complete it (43). It further incentivizes participants to complete self-sampling by promoting the recovery of paid fees, improving the feedback rate and increasing attention to the detection process (44).

This study will focus on women in the high-risk age group for cervical cancer, especially those who have never undergone HPV-Based Self-Sampling or cervical cancer screening. We will use a randomized controlled trial (RCT) design to improve the feedback rate of HPV self-sampling results through prepaid intervention and explore the feasibility and effectiveness of HPV-Based Self-Sampling in cervical cancer screening. The impact of prepaid intervention on HPV positivity rates and subsequent diagnosis and treatment compliance will also be investigated. Further research is needed on factors influencing the improvement of feedback rates through new prepaid interventions, such as participants’ age, education level, and income level. Additionally, evaluating participants’ satisfaction with the entire HPV self-test intervention process can provide insights into the acceptance of the measures during implementation, further enhancing women’s awareness of cervical health and promoting the application of HPV-Based Self-Sampling in female cervical cancer screening, providing a scientific basis.

## 7 Methods

### 7.1 Objective

The primary objective of this study is to evaluate the effectiveness of a Pay-It-Forward approach compared to free distribution of HPV self-sampling kits in improving the feedback rate of HPV self-testing among women in China. We hypothesize that the Pay-It-Forward intervention group will achieve a significantly higher HPV self-testing feedback rate than the free distribution control group. Additionally, we aim to assess whether the Pay-It-Forward intervention leads to higher HPV positivity rates and better adherence to subsequent diagnostic and treatment procedures compared to the control group.

### 7.2 Trial design

**Figure 1:**
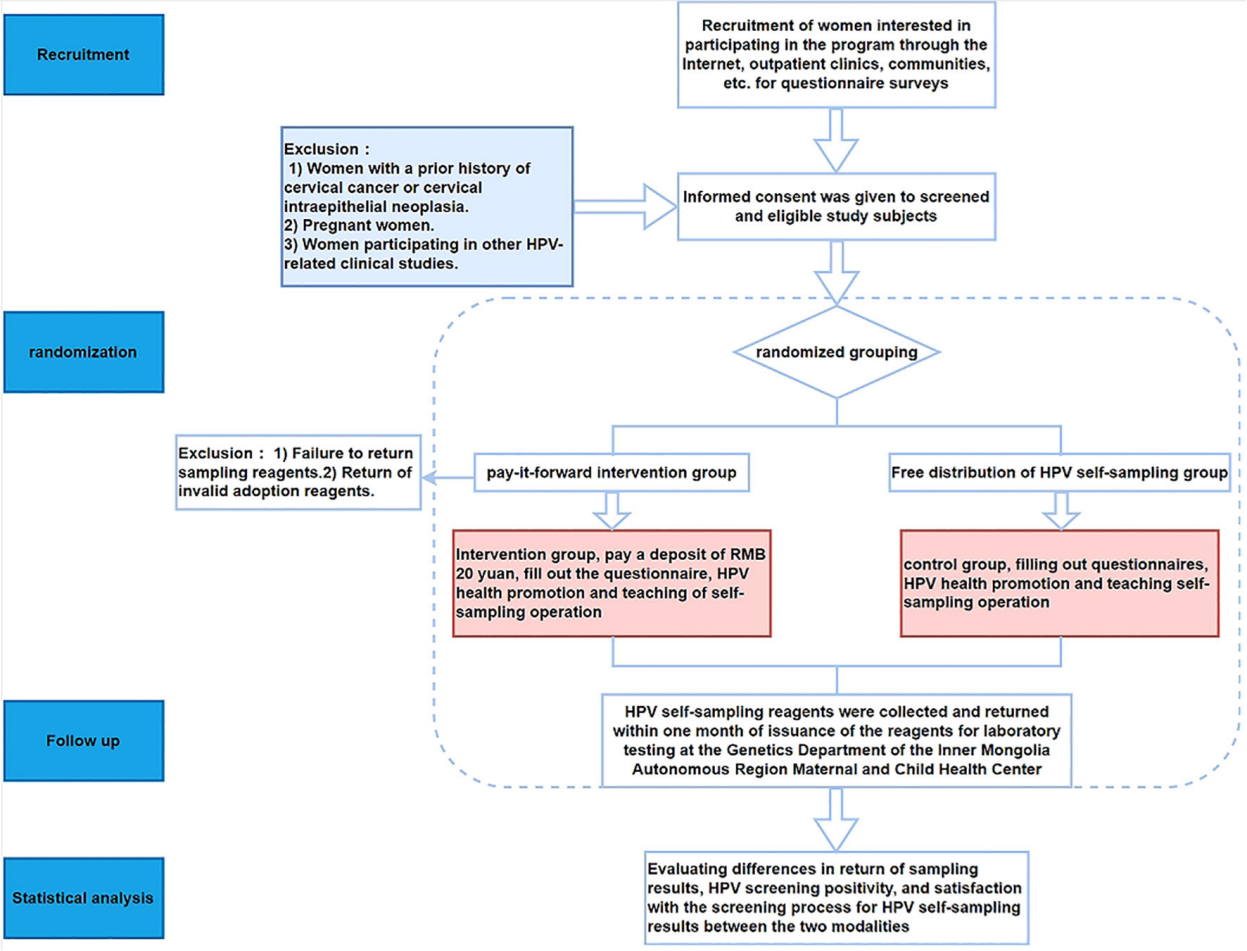
Flowchart.

This study adopts a randomized controlled trial design and is conducted at the Inner Mongolia Maternal and Child Health Care Hospital, Inner Mongolia Autonomous Region, China. The intervention involves implementing a Pay-It-Forward deposit system for women undergoing HPV self-sampling. A total of 108 participants are enrolled and randomly assigned into two groups: the intervention group with 54 participants receiving HPV self-sampling kits through prepayment of a deposit, and the control group with 54 participants receiving HPV self-sampling kits for free. **Figure 1** presents the detailed flowchart of this study. The details of the research process are shown in **Figure 1**.

### 7.3 Recruitment

Participants will be recruited at the Maternal and Child Health Family Planning Service Centers in four urban districts (Xincheng District, Saihan District, Huimin District, and Yuquan District) and five counties (Wuchuan County, Qing Shui He County, Helin County, Tuoketuo County, and Tuo Zuo Banner) of Hohhot City, Inner Mongolia Autonomous Region, China, starting from January 20, 2025.A multi-channel recruitment approach will be employed, including online WeChat public accounts, hospital outpatient clinics, and community health service centers. After identifying eligible and interested individuals, physicians at each Maternal and Child Health Care and Family Planning Service Center further explained the study objectives and procedures. Subsequently, eligible women were screened and selected based on predefined criteria. Each participant received a detailed informed consent form ((refer to Appendix), which outlined the study’s objectives, procedures, and potential implications. Women who reached mutual agreement and signed the informed consent form constituted the recruited cohort. Participants will be then randomly assigned to either the intervention group (“Pay-It-Forward” group) or the control group (“free”group).

### 7.4 Eligibility criteria

The inclusion criteria for this perinatal maternity study include: (1) Women aged 24 years or older; (2) Hold Chinese nationality and have resided in Hohhot for at least six months; (3) Have not undergone HPV self-sampling or cervical cancer screening in the past 12 months; (4) Have not received the HPV vaccine; (5) Possess a mobile phone number and are able to use a smartphone; (6) Are capable of understanding and signing the informed consent form; (7) Without any severe physical or mental illnesses and are able to complete the entire study process. Exclusion criteria are: (1) Women with a history of cervical cancer or cervical intraepithelial neoplasia (CIN); (2) Pregnant women; (3) In other clinical studies related to HPV.

### 7.5 Sample size

The primary outcome is the feedback rate of HPV self-sampling. According to the results of previous studies (9), we hypothesize that the “Pay-It-Forward” intervention will increase the feedback rate to 90%, the feedback rate of HPV self-sampling results in the control group is 61.7%. Using the PASS 15.0 software and applying a two-sided test with α = 0.05 and a power of the test (1 - β) = 0.80, the required sample size per group was calculated to be 86 participants. Considering a potential dropout rate of 20%, we ultimately determined that 54 participants should be recruited per group, resulting in a total sample size of 108 participants.

### 7.6 Randomization

After completing the baseline study visit, participants were randomly allocated to one of the two study groups using a 1:1 ratio. The randomization procedure utilized a random number table approach. The detailed steps are as follows:

1. Random Number Generation: A basic randomization technique was employed. The study designer created a sequence of random numbers using the RANDBETWEEN function in Excel.
2. Sealed Envelopes: These random number sequences were placed in identical, sealed, opaque envelopes that were sequentially numbered. Both the participants and the interveners are unaware of the participants’ group assignments.
3. Enrollment Process: Upon enrollment of each participant, the researcher sequentially opened the envelopes in accordance with a predefined procedure.
4. Assignment: Based on the number revealed in the opened envelope, the participant was assigned to the corresponding study group, and the researcher documented the assignment outcome.

### 7.7 Interventions

**Figure 2:**
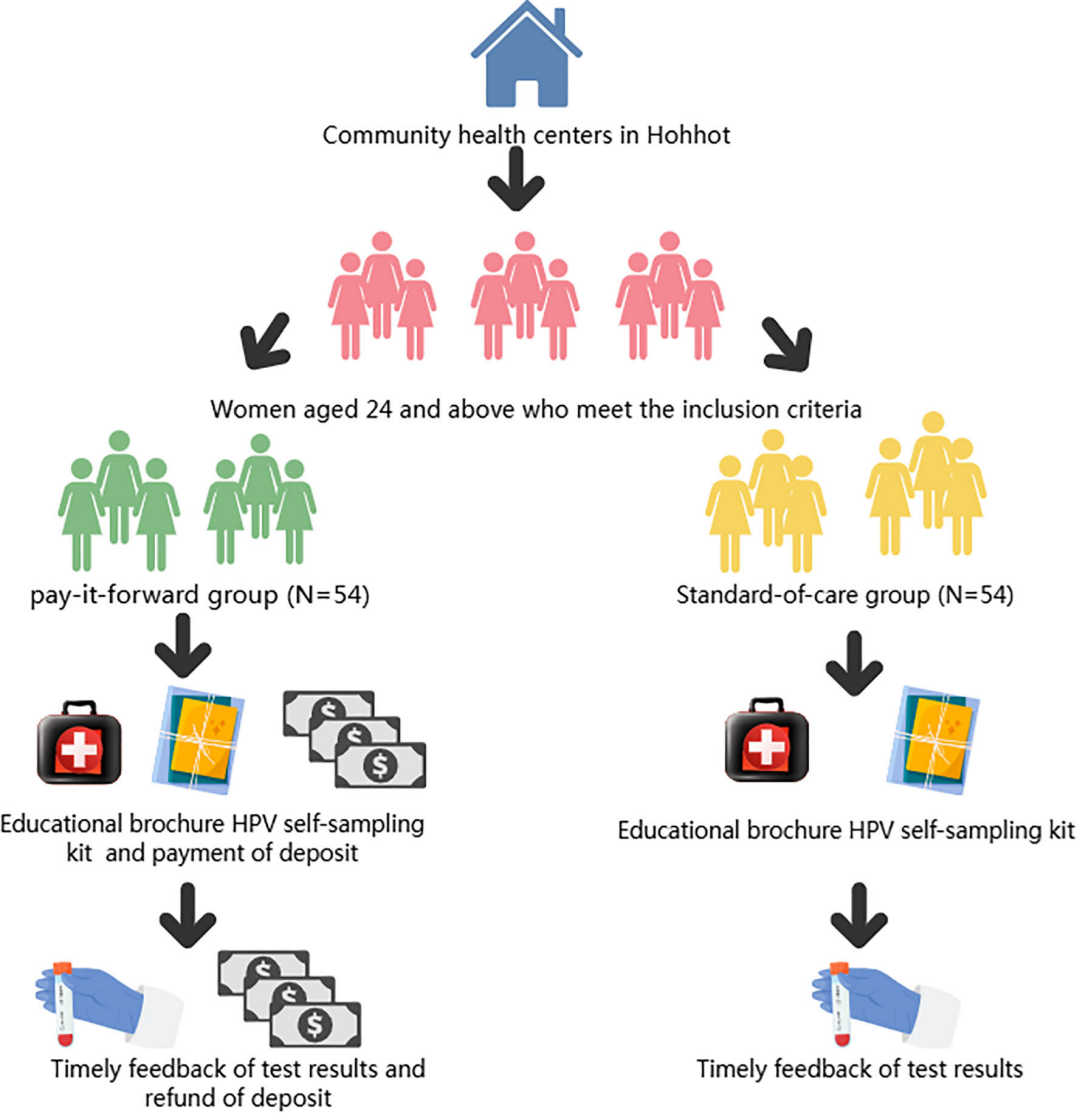
The intervention flow chart.

Compared with the control group, the intervention frequency is only once. That is, when distributing the HPV self-sampling kit, the intervention group needs to prepay to obtain the kit, while the control group can receive the HPV self-sampling kit without payment. Considering that the market price of an HPV self-sampling kit is around 20 RMB, we set the prepayment fee at 20 RMB. This can well simulate the real situation when the participants purchase the kit by themselves in daily life. Moreover, a proper deposit can avoid the problems caused by a high deposit: ① exceeding the participants’ affordability and thus reducing their willingness to participate; ② increasing the participants’ psychological pressure and causing unnecessary worries, which may affect the accuracy of the study results. On the other hand, a proper deposit can also avoid the problems caused by a low deposit: lack of effective constraints, leading to participants not taking the trial seriously and thus causing unstandardized sampling operations and other problems that may affect the trial results. We use the deposit as an incentive to encourage participants to actively provide feedback on the results of HPV self-sampling and to increase the feedback rate of HPV self-sampling results. The intervention flow chart is shown in **Figure 2**. To enhance participants’ psychological commitment and ensure active participation, the full prepayment amount will be refunded upon completion and feedback of the self-sampling results. Participants can choose from various convenient payment methods, including cash, bank transfer, or third-party payment platforms, to minimize payment costs and barriers. After self-sampling, participants in the intervention group can return the sampling kit to the corresponding Maternal and Child Health Care and Family Planning Service Center, which will then forward it to the Genetic and Eugenics Office of the Inner Mongolia Maternal and Child Health Care Hospital. A dedicated hotline will be established to facilitate timely feedback from participants. For those with positive HPV results, free follow-up medical services, including physician consultations and examination appointments, will be provided to ensure they receive proper treatment. Additionally, ongoing monitoring of participants’ adherence to treatment will be conducted.

### 7.8 “Pay-it-forward” collection process

Participants in the intervention group will pay a 20 RMB deposit before receiving the HPV self-sampling kit. This deposit serves as an economic incentive to complete the self-sampling and provide feedback. Staff will be trained on the collection process, record-keeping, and privacy protection. Payments will be accepted via WeChat and Alipay using QR code transfers. Each transaction will be recorded with participant details and verified daily against the number of participants and total amount received. Deposits will be held in a dedicated account, managed by a financial officer who will provide regular reports. Electronic receipts will be issued to participants. A supervisory committee will conduct regular checks to ensure transparency and compliance. In emergencies, collection will be paused, and funds will be securely stored for future refund.

### 7.9 “Pay-it-forward” refund process

Participants will receive a full refund of their deposit after completing the HPV self-sampling and providing feedback. The project team will verify the feedback before initiating the refund process. Once approved, the refund will be processed by the financial officer and returned to the participant via the original payment method. Refund details will be meticulously recorded and verified by financial staff. Electronic refund certificates will be provided to participants. The supervisory committee will oversee the refund process to ensure transparency and compliance. In emergencies, refunds will be paused, and any unreturned funds will be refunded within one month after the study concludes.

### 7.10 Control group

Participants will receive HPV self-sampling kits free of charge. Staff will provide a detailed explanation of the self-sampling procedure and precautions on-site and offer user manuals as well as video or illustrated guides to ensure that participants understand the correct self-sampling method. After completing the self-sampling, participants are required to send the samples to a designated laboratory for HPV testing. The study will cover all costs associated with sample transportation and testing, with no expenses incurred by the participants. Test results will be promptly communicated to participants via phone or text message. For those with positive HPV results, the study will provide information on follow-up diagnosis and treatment and referral channels to assist them in completing further medical examinations and treatment.

### 7.11 Outcomes evaluation

#### 7.11.1 Primary outcome

The primary outcome of this study is the feedback rate of HPV-based self-sampling. It refers to the percentage of participants who mail back the tested HPV sampling kits to us within 1 month after receiving the HPV self-sampling kits, relative to the total number of HPV self-sampling kits distributed. If the feedback is not received within 1 month after distribution, it is considered as no feedback by default.

#### 7.11.2 Secondary outcomes

##### 7.11.2.1 HPV positivity rate and subsequent treatment adherence

The HPV positivity rate, defined as the proportion of participants testing positive for HPV through self-sampling, will be calculated based on laboratory-confirmed results using standardized PCR techniques. Data will be collected from certified laboratories, with a subset of samples retested for validation. Subsequent treatment adherence will be assessed by tracking participants’ attendance at recommended follow-up appointments and receipt of appropriate treatments following a positive HPV result. This will be monitored through electronic medical records and phone follow-ups at 1 and 3 months post-notification. Adherence will be recorded as a binary outcome and expressed as a percentage of participants who follow through with recommended care.

##### 7.11.2.2 Willingness to accept HPV self-sampling

Types of self-sampling tools participants are familiar with, whether they have previously used self-sampling methods for health monitoring, acceptance of the self-sampling intervention concept, and self-assessed ability to perform the self-sampling process. This aspect aims to understand factors hindering women from using self-sampling kits for HPV testing. Factors may include concerns about the accuracy of self-sampling, discomfort or lack of confidence in performing the sampling process, and reluctance to engage in self-sampling due to cultural or personal preferences. Additionally, barriers such as limited access to follow-up healthcare services, lack of awareness about the importance of HPV screening, and logistical challenges in returning the samples to the laboratory will also be explored.

Wu Xueqin’s self-administered questionnaire: Cervical Cancer Knowledge Questionnaire was used to investigate the subjects’ knowledge of cervical cancer, which consisted of 15 questions, all of which were single-choice questions, of which the first question was a jumping question, and if you chose “no”, you did not need to answer the second to fifteenth questions.1 point was scored for correctly answering questions 2-15, and 0 point for wrongly choosing or not knowing, out of 14 points. Questions 2–15 were scored as “1” if they were correctly selected, and “0” if they were incorrectly selected or not known, with a full score of 14. A score of ≥8 was defined as knowledge of cervical cancer according to the cut-off of 60% of the total score (10*60% = 8.4 points, rounded up to 8 points), with the rate of knowledge = number of people who were aware of the knowledge/number of people who responded to the questions*100%.Wu Xueqin’s self-administered questionnaire: Cervical Cancer Screening and HPV Vaccination Attitude Scale was used to investigate subjects’ attitudes toward cervical cancer screening and HPV vaccination, which was measured using a 5-point Likert scale, with scores of 5, 4, 3, 2, and 1 corresponding to “Strongly Agree”,”Agree “, “Can’t say”, “Disagree”, and “Strongly Disagree”, respectively; negative scoring was applied to the barrier perception dimension, i.e. scores 1, 2, 3, 4, and 5 correspond to “strongly agree”, “agree”, “can’t say”, “disagree”, and “strongly disagree”, respectively. The higher the score, the more positive the attitude towards screening or vaccination. Screening rate = number of people screened/number of people in the group participating in the survey*100%, and the same for vaccination rate.

##### 7.11.2.3 Psychological and behavioral health

To assess the severity of anxiety symptoms experienced by participants over the past two weeks, the Generalized Anxiety Disorder-3 (GAD-3) scale will be utilized. This is a shortened version of the GAD-7, comprising three items rated on a four-point scale (0 = “not at all”, 1 = “several days”,2 = “more than half the days”, 3 =“nearly every day”). Total scores range from 0 to 9, with higher scores indicating greater anxiety levels (0-2 = no significant anxiety, 3-5 = mild anxiety, 6-7 = moderate anxiety, 8-9 = severe anxiety).

The Perceived Stress Scale (PSS) will evaluate the degree of perceived stress in participants’ lives, particularly in situations they find unpredictable, uncontrollable, or overloaded. The scale includes four items, each rated on a five-point Likert scale (0 = “never” to 4 = “very often”). Two items are scored as forward scoring questions (1, 4), and two as reverse scoring questions (2, 3). Higher scores reflect higher perceived stress, with a Cronbach’s alpha coefficient of 0.754.

The Family Health Scale Short-Form (FHS-SF) will measure maternal family health functioning across four dimensions: family/social/emotional health processes, family healthy lifestyle, family health resources, and external social support. The scale consists of 10 items, each scored on a five-point scale (1 = “strongly disagree” to 5 = “strongly agree”), with items 6, 9, and 10 reverse scored. Higher total scores indicate better maternal family health, with a Cronbach’s alpha coefficient of 0.846.

Health literacy will be assessed using the Health Literacy Scale (HIS), a four-item multiple-choice questionnaire divided into three dimensions: health care, disease prevention, and health promotion. Responses are categorized as very easy, easy, difficult, or very difficult.

The New General Self-Efficacy Scale (NGSES-SF) will measure psychological dimensions across three aspects: level or degree, intensity, and prevalence, with response options ranging from strongly disagree to strongly agree.

Social support levels will be evaluated using the Perceived Social Support Scale-Short Form (PSSS-SF), a three-item version of the original PSSS. This scale assesses overall perceived social support from family, friends, and significant others on a 7-point Likert scale (1 = “not at all compatible” to 7 = “fully compatible”). Total scores range from 3 to 21, with higher scores indicating greater perceived social support (3-9 = low support, 10-17 = moderate support, 18-21 = high support).

Personality traits will be assessed using the Chinese Big Five Personality Inventory Brief Version (CBF-PI-B), which evaluates five dimensions: neuroticism, extraversion, openness, agreeableness, and conscientiousness. Each dimension is represented by a single item rated on a 6-point scale (1 = “not at all” to 6 = “fully”), providing a quick assessment of key personality traits.

Depression severity will be measured using the Patient Health Questionnaire-9 (PHQ-9), a nine-item tool corresponding to DSM-5 criteria for major depressive disorder. Each item assesses the frequency of depressive symptoms over the past two weeks on a 4-point scale (0 = “not at all” to 3 = “nearly every day”). Total scores range from 0 to 27, with higher scores indicating greater depression severity (0-4 = minimal depression, 5-9 = mild depression, 10-14 = moderate depression, 15-19 = moderately severe depression, 20-27 = severe depression).

### 7.12 Follow-up

This is a six-month study and the intervention will be implemented during the first four weeks of subject enrollment. All subjects participating (both the “Pay-It-Forward” group and control group) are required to finish electronic questionnaires at two time points, including baseline and post intervention. The content of the follow-up visits varied across time points, with sociodemographic information and baseline health data collected only at baseline. Behavioral information, HPV self-sampling results, and participant satisfaction data will be collected at the 4-week post-intervention follow-up point. The specific follow-up and related data collection schedule is shown in **Table 1**.

**Table 1:** Variables and measures collected at every time points.

Variables		Date source	Study Period	Baseline (T0)	Post-intervention (T1)
			timepoint	0w ± 1w	4w ± 1w
Intervention preparation	\	\	Informed consent	✓	\
	\	\	Random grouping	✓	\
	\	\	Online consulting service	✓	✓
Covariates	Individual	Baseline questionnaire	Sociodemographic information	✓	\
	Individual	Baseline questionnaire and Follow-up questionnaire	Behavioral information	✓	✓
Primary outcome	Feedback rate of HPV self-sampling	Laboratory records	Percentage of participants who return the HPV self-sampling kit within 1 month	\	✓
HPV infection status (Secondary Outcome)	HPV positivity rate	Laboratory records	Proportion of participants with lab-confirmed HPV-positive results (PCR testing)	\	✓
HPV self-sampling cognition and acceptance (Secondary Outcome)	HPV Self-Sampling Awareness	Follow-up questionnaire	HPV Self-Sampling Awareness Questionnaire	\	✓
	HPV self-sampling Satisfaction	Follow-up questionnaire	HPV self-sampling Satisfaction Questionnaire	\	✓
	HPV Self-Sampling Acceptance	Follow-up questionnaire	HPV Self-Sampling Acceptance Scale	\	✓
Cervical cancer health literacy and attitudes (Secondary Outcome)	Cervical Cancer-related Knowledge	Baseline questionnaire	Cervical Cancer-related Knowledge Scale	✓	\
	Cervical Cancer Screening and HPV Vaccination Attitude	Baseline questionnaire	Cervical Cancer Screening and HPV Vaccination Attitude Scale	✓	\
Psychology status (Secondary Outcomes)	depressed	Baseline questionnaire	PHQ-9	✓	\
	Anxiety	Baseline questionnaire	GAD-3	✓	\
	Stress	Baseline questionnaire	PSS-4	✓	\
	Family health	Baseline questionnaire	FHS-SF	✓	\
	Social support	Baseline questionnaire	PSSS-SF	✓	\
	personalities	Baseline questionnaire	BFI	✓	\
Health status(Secondary Outcomes)	health awareness	Baseline questionnaire	HLS	✓	\

The full name of PHQ-9 is Patient Health Questionnaire-9 Scale; The full name of GAD-3 is Generalized Anxiety Disorder scale; The full name of PPS-4 is Perceived Stress Scale; The full name of FHS-SF is The Family Health Scale short-form; The full name is HLS is Health Literacy Scale; The full name of PSSS-SF is Perceived Social Support Scale; The full name of BFI is The big five inventory.

### 7.13 Statistical methods

Upon completion of the trial, all collected case data and questionnaire responses will be collated and summarized. Data storage will be managed using Microsoft Office 2017, while statistical analysis will be conducted using SPSS Statistics 27.0 software. Count data will be presented as the number of cases (N) and percentage (%). The distribution of continuous data will be assessed for normality; normally distributed data will be expressed as mean ± standard deviation, whereas non-normally distributed data will be presented as median (interquartile range) and quartile spacing (P25–P75). Baseline characteristics between the intervention and control groups will be compared using appropriate statistical tests, such as the independent samples t-test, Mann-Whitney U-test, and chi-square test, depending on the data type. Longitudinal analysis of follow-up data will be conducted using Generalized Estimating Equations (GEE) to compare differences in outcomes between the two groups. A two-sided test will be used, with statistical significance set at p < 0.05. In cases of missing data, multiple imputation techniques will be applied, given the random nature of the missing data. Multiple imputation involves generating multiple complete datasets by replacing missing values with plausible estimates derived from observed data and imputation models. This method preserves dataset variability and enhances result reliability compared to single imputation methods. Multiple imputation will be implemented using established statistical software like SPSS or R. Sensitivity analyses will also be conducted to assess the robustness of the results under various assumptions about missing data. This comprehensive approach ensures the validity and reliability of the study findings, accounting for missing data while maintaining statistical integrity throughout the analysis.

### 7.14 Collection and archiving of data

During the trial, we will use the Questionnaire Star platform to collect survey data from all participants at baseline and during follow-up visits. These questionnaires can be accessed and completed online via a QR code, and they will collect information from participants, including sociodemographic and behavioral data. After the study concludes, the researchers will systematically organize and store all study-related data and documents, including exported questionnaires, group allocation records, follow-up information, laboratory test results, informed consent forms, and so on, into a database designated for this study. All raw data and documents will be archived in accordance with relevant laws and regulations after the trial ends.

### 7.15 Investigator responsibilities

In this study, the primary responsibilities of the researchers include ensuring the scientific validity and rationality of the study design, protecting the rights of participants, ensuring the authenticity of data, and supervising the implementation of the study according to the research protocol. Prior to the commencement of the trial, researchers will train team members to ensure their familiarity with the study protocol and data collection procedures. During the study, regular supervision of team members’ fieldwork and documentation will be conducted.

Additionally, researchers will closely monitor and document any adverse events (AEs) experienced by participants during the intervention period, including psychological reactions, mood changes, behavioral alterations, and difficulties in using technology. Documentation will include the severity of the event, time of occurrence, duration, interventions taken, and final outcomes. Any serious adverse events (SAEs) identified will be reported in accordance with the regulations and timelines set by the Medical Ethics Committee of the Inner Mongolia Maternal and Child Health Hospital. For urgent SAEs, the study team will immediately implement necessary medical measures and promptly notify the ethics committee and sponsor.

### 7.16 Data monitoring

A Data Monitoring Committee (DMC) will be established for this study, comprising at least two members from the Medical Ethics Committee of the Inner Mongolia Maternal and Child Health Hospital. Operating independently of the study team, the DMC will regularly assess trial progress and safety data according to the established Data Monitoring Plan (DMP). The scope of monitoring will cover questionnaire data, monitoring results, and records of deposits and refunds. The primary responsibilities of the DMC are to ensure data integrity, evaluate participant safety, and protect participant rights. All monitoring activities will be conducted in a blinded manner to ensure data objectivity and the reliability of trial results. The DMC has the authority to immediately suspend or terminate the study if any non-compliance with the approved protocol or unauthorized changes in study procedures are detected. The Principal Investigator, Co-Investigators, and designated personnel from the Sponsor’s organization will have access to the final trial dataset. Additionally, external auditors, in addition to the Data Monitoring Committee, may also be granted access.

## 8 Discussion

HPV-based self-sampling has emerged as a promising approach to increase cervical cancer screening coverage, especially in hard-to-reach populations (1). However, a critical challenge remains: the low feedback rate of self-sampling results, which undermines the effectiveness of screening programs (2). Enhancing the feedback rate of HPV self-sampling results is crucial for ensuring that women receive timely diagnosis and treatment, thus improving the overall efficacy of cervical cancer screening (3, 45). Pay-It-Forward interventions have shown potential in improving screening effectiveness by leveraging the psychological effect of commitment (4, 45). This study will explore the application of a Pay-It-Forward approach to increase the feedback rate of HPV self-sampling results, aiming to fill the gap in current screening strategies (1).

This study pioneers the application of the Pay-It-Forward method, a novel economic incentive approach, to improve the feedback rate of HPV self-sampling results. By requiring participants to pre-pay a certain fee, which is refunded upon completion of self-sampling and feedback of results, this model is expected to enhance participants’ motivation and sense of responsibility (46). This psychological commitment effect may lead to a higher feedback rate compared to traditional screening interventions (10, 47). Another innovation of this study lies in its extensive geographical representation and in-depth urban-rural disparity analysis. By covering all urban districts and surrounding counties of the city and equally recruiting participants from each region, we ensure the generalizability and fairness of the study results (15, 48). This design not only reduces selection bias but also promotes health equity by providing equal health intervention opportunities for women in different areas (16).

The Pay-It-Forward intervention is expected to be a feasible and innovative approach to improve screening participation and feedback rates. This approach may have a significant impact on cervical cancer screening-related public health policies by increasing the number of women who complete the screening process. The study also highlights the importance of addressing health inequalities by promoting more effective screening strategies that are accessible to women in ethnic minority regions. In the global effort to eliminate cervical cancer, this study seeks to contribute to international guidelines and strategies for HPV self-sampling screening. By providing evidence on the effectiveness of economic incentives in improving screening outcomes, it may inform the development of culturally-appropriate and effective screening programs worldwide.

A potential limitation of this study is the relatively small sample size, which may affect the statistical power and generalizability of the findings. Future research should consider larger sample sizes to validate the results and ensure broader applicability. Additionally, we acknowledge a potential selection bias in participant recruitment that may limit the generalizability of our findings to populations with lower health consciousness.

Future studies should explore different prepayment amounts and methods to determine the optimal approach for maximizing the feedback rate. Additionally, research on the cost-effectiveness of implementing Pay-It-Forward interventions in resource-limited settings is needed to inform practical applications. Based on the expected results, specific recommendations for culturally-adapted interventions and community engagement should be provided. These recommendations can guide the development of targeted screening programs that are sensitive to the needs and contexts of ethnic minority women. The potential application of similar economic incentive models to other health screening programs should also be discussed. This may include exploring how such models can be adapted to address other health issues and promote screening participation in diverse populations.

The findings of this study will highlight the potential of innovative economic incentives, such as the Pay-It-Forward approach, in enhancing the feedback rate of HPV self-sampling and improving overall screening effectiveness. These results will underscore the critical role of community engagement and culturally tailored interventions in addressing health disparities among ethnic minority populations. We advocate for the continued exploration of novel strategies in cervical cancer screening to ensure equitable access and improved health outcomes. Ultimately, this study supports the broader application of evidence-based interventions to drive meaningful progress in public health initiatives.

## Ethics statement

This study adheres to the ethical principles outlined in the GCP and the current revised Declaration of Helsinki. It complies with all relevant legal and regulatory requirements of the trial location. Approval has been obtained from the Ethics Committee of the Inner Mongolia Maternal and Child Health Care Hospital ([2023] Lun Han Shen No.[094-1]) and registered on ClinicalTrials.gov (ChiCTR2500095770) as of January 13, 2025. Any protocol modifications requiring formal approval will be communicated to the Ethics Committee. The committee will be notified of the trial’s completion. Informed consent will be obtained from all participants prior to their involvement, with clear documentation through signed consent forms. For participants unable to sign, witnessed oral consent will be accepted, ensuring they fully understand the trial’s nature and implications.

## References

[1] Kombe Kombe AJ Li B Zahid A Mengist HM Bounda GA Zhou Y . Epidemiology and burden of human papillomavirus and related diseases, molecular pathogenesis, and vaccine evaluation. Front Public Health. (2020) 8:552028. doi: 10.3389/fpubh.2020.552028 33553082 PMC7855977

[2] de Sanjosé S Diaz M Castellsagué X Clifford G Bruni L Muñoz N . Worldwide prevalence and genotype distribution of cervical human papillomavirus DNA in women with normal cytology: a meta-analysis. Lancet Infect Dis. (2007) 7:453–9. doi: 10.1016/S1473-3099(07)70158-5 17597569

[3] Forman D de Martel C Lacey CJ Soerjomataram I Lortet-Tieulent J Bruni L . Global burden of human papillomavirus and related diseases. Vaccine. (2012) 30 Suppl 5:F12–23. doi: 10.1016/j.vaccine.2012.07.055 23199955

[4] Guan P Howell-Jones R Li N Bruni L de Sanjosé S Franceschi S . Human papillomavirus types in 115,789 HPV-positive women: a meta-analysis from cervical infection to cancer. Int J Cancer. (2012) 131:2349–59. doi: 10.1002/ijc.v131.10 22323075

[5] Li Y Chen S Huang X Fang Y Zhao Q . Clinical utility and economic evaluation of human papillomavirus vaccines. Modern Prevent Med. (2018) 45(15):2840–3.

[6] Zhu B Liu Y Zuo T Cui X Li M Zhang J . The prevalence, trends, and geographical distribution of human papillomavirus infection in China: The pooled analysis of 1.7 million women. Cancer Med. (2019) 8:5373–85. doi: 10.1002/cam4.v8.11 PMC6718589 31350872

[7] Zhang J Cheng K Wang Z . Prevalence and distribution of human papillomavirus genotypes in cervical intraepithelial neoplasia in China: a meta-analysis. Arch Gynecol Obstet. (2020) 302:1329–37. doi: 10.1007/s00404-020-05787-w PMC7584548 32914222

[8] Brisson M Kim JJ Canfell K Drolet M Gingras G Burger EA . Impact of HPV vaccination and cervical screening on cervical cancer elimination: a comparative modelling analysis in 78 low-income and lower-middle-income countries. Lancet. (2020) 395:575–90. doi: 10.1016/S0140-6736(20)30068-4 PMC7043009 32007141

[9] Winer RL Lin J Anderson ML Tiro JA Green BB Gao H . Strategies to increase cervical cancer screening with mailed human papillomavirus self-sampling kits: A randomized clinical trial. Jama. (2023) 330:1971–81. doi: 10.1001/jama.2023.21471 PMC10685881 38015219

[10] Xia C Xu X Zhao X Hu S Qiao Y Zhang Y . Effectiveness and cost-effectiveness of eliminating cervical cancer through a tailored optimal pathway: a modeling study. BMC Med. (2021) 19:62. doi: 10.1186/s12916-021-01930-9 33653331 PMC7927373

[11] Curry SJ Krist AH Owens DK Barry MJ Caughey AB Davidson KW . Screening for cervical cancer: US preventive services task force recommendation statement. Jama. (2018) 320:674–86. doi: 10.1001/jama.2018.10897 30140884

[12] Peirson L Fitzpatrick-Lewis D Ciliska D Warren R . Screening for cervical cancer: a systematic review and meta-analysis. Syst Rev. (2013) 2:1–14. doi: 10.1186/2046-4053-2-35 23706117 PMC3681632

[13] Perkins RB Guido RS Castle PE Chelmow D Einstein MH Garcia F . 2019 ASCCP risk-based management consensus guidelines for abnormal cervical cancer screening tests and cancer precursors. J Low Genit Tract Dis. (2020) 24:102–31. doi: 10.1097/LGT.0000000000000525 PMC7147428 32243307

[14] Harris RP Helfand M Woolf SH Lohr KN Mulrow CD Teutsch SM . Current methods of the US Preventive Services Task Force: a review of the process. Am J Prev Med. (2001) 20:21–35. doi: 10.1016/S0749-3797(01)00261-6 11306229

[15] Organization WH . WHO guidelines for screening and treatment of precancerous lesions for cervical cancer prevention: supplemental material: GRADE evidence-to-recommendation tables and evidence profiles for each recommendation. Geneva: World Health Organization (2013). 24716265

[16] Daponte N Valasoulis G Michail G Magaliou I Daponte AI Garas A . HPV-based self-sampling in cervical cancer screening: an updated review of the current evidence in the literature. Cancers (Basel). (2023) 15:1669. doi: 10.3390/cancers15061669 36980555 PMC10046242

[17] Aarnio R Östensson E Olovsson M Gustavsson I Gyllensten U . Cost-effectiveness analysis of repeated self-sampling for HPV testing in primary cervical screening: a randomized study. BMC Cancer. (2020) 20:645. doi: 10.1186/s12885-020-07085-9 32660432 PMC7359275

[18] Szarewski A Cadman L Mallett S Austin J Londesborough P Waller J . Human papillomavirus testing by self-sampling: assessment of accuracy in an unsupervised clinical setting. J Med Screen. (2007) 14:34–42. doi: 10.1258/096914107780154486 17362570 PMC4109399

[19] Costa S Verberckmoes B Castle PE Arbyn M . Offering HPV self-sampling kits: an updated meta-analysis of the effectiveness of strategies to increase participation in cervical cancer screening. Br J Cancer. (2023) 128:805–13. doi: 10.1038/s41416-022-02094-w PMC9977737 36517552

[20] Mullins R Scalzo K Sultana F . Self-sampling for cervical screening: could it overcome some of the barriers to the Pap test? J Med Screen. (2014) 21:201–6. doi: 10.1177/0969141314555247 25312640

[21] Bansil P Wittet S Lim JL Winkler JL Paul P Jeronimo J . Acceptability of self-collection sampling for HPV-DNA testing in low-resource settings: a mixed methods approach. BMC Public Health. (2014) 14:596. doi: 10.1186/1471-2458-14-596 24927941 PMC4061776

[22] Wong LP Han L Li H Zhao J Zhao Q Zimet GD . Current issues facing the introduction of human papillomavirus vaccine in China and future prospects. Hum Vaccin Immunother. (2019) 15:1533–40. doi: 10.1080/21645515.2019.1611157 PMC6746483 31017500

[23] Serrano B Ibáñez R Robles C Peremiquel-Trillas P de Sanjosé S Bruni L . Worldwide use of HPV self-sampling for cervical cancer screening. Prev Med. (2022) 154:106900. doi: 10.1016/j.ypmed.2021.106900 34861338

[24] Serrano B Ibañez R Robles C Peremiquel-Trillas P de Sanjose S . Worldwide use of HPV self-sampling for cervical cancer screening. Prev Med. (2022) 154:106900. doi: 10.1016/j.ypmed.2021.106900 34861338

[25] Guo C Du H Qu X Duan X Li J Li R . Prevalence of human papillomavirus among Chinese Han and Mongols minority women in inner Mongolia, China: reflected by self-collected samples in CHIMUST. Front Public Health. (2022) 10:840879. doi: 10.3389/fpubh.2022.840879 35692337 PMC9174663

[26] Yang H Li SP Chen Q Morgan C . Barriers to cervical cancer screening among rural women in eastern China: a qualitative study. BMJ Open. (2019) 9:e026413. doi: 10.1136/bmjopen-2018-026413 PMC6429857 30872552

[27] Song J Ni YH Fang J Qu SX Chen XY Wu WL . The levels of women’s awareness, experience, acceptability and preference for Vaginal Human Papillomavirus (HPV) self-sampling in three provinces of China: a cross-sectional study. BMC Womens Health. (2024) 24:343. doi: 10.1186/s12905-024-03186-w 38877469 PMC11179292

[28] Horner-Johnson W Dobbertin K Iezzoni LI . Disparities in receipt of breast and cervical cancer screening for rural women age 18 to 64 with disabilities. Womens Health Issues. (2015) 25:246–53. doi: 10.1016/j.whi.2015.02.004 25864023

[29] Lin CY Chen HC Lin RW You SL You CM Chuang LC . Quality assurance of genotyping array for detection and typing of human papillomavirus. J Virol Methods. (2007) 140:1–9. doi: 10.1016/j.jviromet.2006.10.004 17118466

[30] Vega-Crespo B Neira VA Maldonado-Rengel R López D Delgado-López D Guerra Astudillo G . Barriers and advantages of self-sampling tests, for HPV diagnosis: A qualitative field experience before implementation in a rural community in Ecuador. Int J Womens Health. (2024) 16:947–60. doi: 10.2147/IJWH.S455118 PMC11143988 38827925

[31] Zhang B Wang S Yang X Chen M Ren W Bao Y . Knowledge, willingness, uptake and barriers of cervical cancer screening services among Chinese adult females: a national cross-sectional survey based on a large e-commerce platform. BMC Womens Health. (2023) 23:435. doi: 10.1186/s12905-023-02554-2 37592252 PMC10436426

[32] Zhetpisbayeva I Kassymbekova F Sarmuldayeva S Semenova Y Glushkova N . Cervical cancer prevention in rural areas. Ann Glob Health. (2023) 89:75. doi: 10.5334/aogh.4133 37928103 PMC10624144

[33] Shin MBY . Cervical Cancer Elimination in Low-and-middle-income countries: The Role of Cost and Empowerment in the Implementation of Human Papillomavirus Self-Sampling. Seattle: University of Washington (2021).

[34] Li J Wu R Qu X Huang X Li L Lin Z . Effectiveness and feasibility of self-sampling for human papillomavirus testing for internet-based cervical cancer screening. Front Public Health. (2022) 10:938272. doi: 10.3389/fpubh.2022.938272 36420004 PMC9677822

[35] Nishimura H Yeh PT Oguntade H Kennedy CE Narasimhan M . HPV self-sampling for cervical cancer screening: a systematic review of values and preferences. BMJ Glob Health. (2021) 6:e003743. doi: 10.1136/bmjgh-2020-003743 PMC8137189 34011537

[36] Asare M Lanning BA Isada S Rose T Mamudu HM . Feasibility of utilizing social media to promote HPV self-collected sampling among medically underserved women in a rural southern city in the United States (U.S.). Int J Environ Res Public Health. (2021) 18:10820. doi: 10.3390/ijerph182010820 34682565 PMC8535372

[37] Madzima TR Vahabi M Lofters A . Emerging role of HPV self-sampling in cervical cancer screening for hard-to-reach women: Focused literature review. Can Fam Physician. (2017) 63:597–601. doi: 10.2147/IJWH.S455118 28807952 PMC5555324

[38] Shin MB Garcia PJ Saldarriaga EM Fiestas JL Ásbjörnsdóttir KH Iribarren SJ . Cost of community-based human papillomavirus self-sampling in Peru: A micro-costing study. Lancet Reg Health Am. (2022) 8:100160. doi: 10.1016/j.lana.2021.100160 35528707 PMC9075528

[39] Caskey R Sherman EG Beskin K Rapport R Xia Y Schwartz A . A behavioral economic approach to improving human papillomavirus vaccination. J Adolesc Health. (2017) 61:755–60. doi: 10.1016/j.jadohealth.2017.07.020 29037471

[40] Ma GX Zhu L Zhai S Lin TR Tan Y Johnson C . Empowering low-income Asian American women to conduct human papillomavirus self-sampling test: A community-engaged and culturally tailored intervention. Cancer Control. (2022) 29:10732748221076813. doi: 10.1177/10732748221076813 35193408 PMC8874186

[41] Jede F Brandt T Gedefaw M Wubneh SB Abebe T Teka B . Home-based HPV self-sampling assisted by a cloud-based electronic data system: Lessons learnt from a pilot community cervical cancer screening campaign in rural Ethiopia. Papillomavirus Res. (2020) 9:100198. doi: 10.1016/j.pvr.2020.100198 32416283 PMC7240728

[42] Buist DSM Tiro JA Thayer C Beatty T Miglioretti DL Lin J . Improving the promise of embedded pragmatic trials: Surmountable barriers encountered in an evaluation of home-based HPV self-sampling to increase cervical cancer screening in overdue women. Contemp Clin Trials Commun. (2019) 15:100413. doi: 10.1016/j.conctc.2019.100413 31372572 PMC6661276

[43] Meenan RT Troja C Buist DSM Tiro JA Lin J Anderson ML . Economic evaluation of mailed home-based human papillomavirus self-sampling kits for cervical cancer screening. JAMA Netw Open. (2023) 6:e234052. doi: 10.1001/jamanetworkopen.2023.4052 36947040 PMC10034577

[44] Nelson EJ Maynard BR Loux T Fatla J Gordon R Arnold LD . The acceptability of self-sampled screening for HPV DNA: a systematic review and meta-analysis. Sex Transm Infect. (2017) 93:56–61. doi: 10.1136/sextrans-2016-052609 28100761

[45] Li KT Tang W Wu D Huang W Wu F Lee A . Pay-it-forward strategy to enhance uptake of dual gonorrhea and chlamydia testing among men who have sex with men in China: a pragmatic, quasi-experimental study. Lancet Infect Dis. (2019) 19:76–82. doi: 10.1016/S1473-3099(18)30556-5 30587296 PMC6347395

[46] Li Y Li J Tucker JD Geng EH Wu D . Pay-it-forward as a strategy to increase vaccine uptake. BMC Glob Public Health. (2025) 3:32. doi: 10.1186/s44263-025-00151-z 40200370 PMC11980111

[47] Yang F Zhang TP Tang W Ong JJ Alexander M Forastiere L . Pay-it-forward gonorrhoea and chlamydia testing among men who have sex with men in China: a randomised controlled trial. Lancet Infect Dis. (2020) 20:976–82. doi: 10.1016/S1473-3099(20)30172-9 PMC8957706 32530426

[48] Marley G Tan RKJ Wu D Wang T Sun M Sheng Q . Pay-it-forward gonorrhea and chlamydia testing among men who have sex with men and male STD patients in China: the PIONEER pragmatic, cluster randomized controlled trial protocol. BMC Public Health. (2023) 23:1182. doi: 10.1186/s12889-023-16095-8 37337181 PMC10280958

