# Supplementary material for: A pay-it-forward approach to improve feedback rate of HPV-based self-sampling in cervical cancer screening among women in ethnic minority regions of China: a randomized controlled trial protocol
Source: Front Psychiatry. 2025 May 29;16:1586076. doi: 10.3389/fpsyt.2025.1586076 (PMC12158934; doi:10.3389/fpsyt.2025.1586076)
Supplement: Supplementary file 1 [file DataSheet1.zip › Datasheet 2.docx]

**Follow-up Questionnaire on Using “Pay-It-Forward” Approach to Improve Feedback Rate of HPV-Based Self-Sampling in Cervical Cancer Screening Among Women in Ethnic Minority Regions of China**

Research objective: We hereby invite you to participate in a survey before you decide to participate in this study, please make sure to confirm the purpose of the study and the issues it will involve Please read the following information carefully. If you have any unclear information or need more information, please consult the researchers.

This study will comprehensively evaluate the effectiveness of prepaid intervention strategies compared to free distribution of HPV self-sampling in improving the feedback rate of HPV self-sampling results among Chinese women.

Research risk: Some questions in the questionnaire may make you feel uncomfortable, but there are no known risks associated with completing this survey You may refuse to answer some or all of the questions If you wish, you can terminate your questionnaire at any time.

The benefits of participating in the survey: In this study, you will gain knowledge about women's health protection and disease prevention. The information you provide, whether it is actual feedback during the sample collection process or suggestions for the research process, will help the research team to deeply analyze the actual effectiveness and potential influencing factors of prepaid intervention measures in improving HPV self-sampling feedback rate This will help optimize the allocation of medical resources, promote the improvement of the public health system, provide key data support and practical experience references for accurately formulating women's health management strategies and enhancing disease prevention and control capabilities, and play an important role in improving women's health levels and promoting the overall healthy development of society.

Confidentiality of the survey: Your answers to this questionnaire will be treated as confidential.

Voluntary participation: You voluntarily participate in this study. It will be up to you to decide whether you will participate in this study. If you decide to participate in this study, you will be required to sign an informed consent form. After signing the consent form, you can still withdraw at any time without reason, and withdrawing from this study will not affect your relationship with the researchers.

Informed Consent Form: I have read and am aware of the information provided, and have the opportunity to raise questions. I voluntarily participate in the survey and can withdraw at any time without reason. If you agree and acknowledge the above content, please click the "Agree" button below to begin answering the questions. If you do not agree or acknowledge the content, please click the "Disagree" button below to interrupt this survey project.

If you agree to participate in our survey, please click on 'Agree' below and start the questionnaire survey.[ Single Choice Question]*

| ○ Agree |
| --- |
| ○ Disagree |

2. Your name: [fill in the blank question]*

_________________________________

3. Your phone number: [fill in the blank question]*

_________________________________

4. How is the completion status of your HPV self-sampling? [Single Choice Question]*

| ○ Complete on time |
| --- |
| ○ Overdue |
| ○ Incomplete |

5. Where do you prefer to complete HPV self-sampling? [Single Choice Question]*

| ○ Hospital |
| --- |
| ○ Nearby clinics |
| ○ At home |
| ○ Other |

6. What are your concerns about the HPV self-sampling screening method? [Multiple Choice Question] *

| □ No concerns |
| --- |
| □Deterioration and contamination during transportation |
| □ Difficulty in sampling operation |
| □ Unsafe sampling (there may be risks of bodily injury, discomfort, or infection caused by improper sampling) |
| □The result is unreliable |
| □ Unable to provide timely feedback on results |
| □ Other |

7. What is the reason for choosing HPV self-sampling? [Multiple Choice Question] *

| □Easy to operate |
| --- |
| □ Not awkward |
| □ Protect privacy |
| □ Relatively comfortable |
| □You can decide the location by yourself |
| □Strong flexibility in time |

8. How difficult do you think HPV self-sampling is? [Single Choice Question]*

| ○ Very simple |
| --- |
| ○ Simple |
| ○ Generally |
| □It's not simple |
| ○ Very not simple |

9. How was your experience with HPV self-sampling this time? [Single Choice Question]*

| ○ Very comfortable |
| --- |
| ○ Comfortable |
| ○ Generally |
| ○ Discomfort |
| ○ Extremely uncomfortable |

10. How accepting are you of the HPV self-sampling method? [Single Choice Question]*

| ○ Fully accepted |
| --- |
| ○ Accept |
| ○ Generally |
| ○ Not accepted |
| ○ Completely unacceptable |

11. How long did it take you from the beginning to the end of sampling [Single choice question]*

| ○<5 minutes |
| --- |
| ○ 5-10 minutes |
| ○>10 minutes |

12. Whether you have bleeding after HPV self-sampling [Single choice question]*

| ○ Yes |
| --- |
| ○ None |

13. Are you satisfied with the HPV self-sampling testing method used in this case? [Single Choice Question]*

| ○ Very satisfied |
| --- |
| ○ More satisfied |
| ○ Generally |
| ○ Less satisfied |
| ○ Very dissatisfied |
