# Supplementary material for: A pay-it-forward approach to improve feedback rate of HPV-based self-sampling in cervical cancer screening among women in ethnic minority regions of China: a randomized controlled trial protocol
Source: Front Psychiatry. 2025 May 29;16:1586076. doi: 10.3389/fpsyt.2025.1586076 (PMC12158934; doi:10.3389/fpsyt.2025.1586076)
Supplement: Supplementary file 1 [file DataSheet1.zip › Datasheet 1.docx]

**Baseline Questionnaire on Using “Pay-It-Forward” Approach to Improve Feedback Rate of HPV-Based Self-Sampling in Cervical Cancer Screening Among Women in Ethnic Minority Regions of China**

Research objective: We invite you to participate in a survey before you decide to participate in this study, please make sure to confirm the purpose of the study and the issues. Please read the following information carefully. If you have any unclear information or need more information, please consult the researchers.

This study will comprehensively evaluate the effectiveness of prepaid intervention strategies compared to free distribution of HPV self-sampling in improving the feedback rate of HPV self-sampling results among Chinese women.

Research risk: Some questions in the questionnaire may make you feel uncomfortable, but there are no known risks associated with completing this survey You may refuse to answer some or all of the questions If you wish, you can terminate your questionnaire at any time.

The benefits of participating in the survey: In this study, you will gain knowledge about women's health protection and disease prevention. The information you provide, whether it is actual feedback during the sample collection process or suggestions for the research process, will help the research team to deeply analyze the actual effectiveness and potential influencing factors of prepaid intervention measures in improving HPV self-sampling feedback rate This will help optimize the allocation of medical resources, promote the improvement of the public health system, provide key data support and practical experience references for accurately formulating women's health management strategies and enhancing disease prevention and control capabilities, and play an important role in improving women's health levels and promoting the overall healthy development of society.

Confidentiality of the survey: Your answers to this questionnaire will be treated as confidential.

Voluntary participation: You voluntarily participate in this study. It will be up to you to decide whether you will participate in this study. If you decide to participate in this study, you will be required to sign an informed consent form. After signing the consent form, you can still withdraw at any time without reason, and withdrawing from this study will not affect your relationship with the researchers.

Informed Consent Form: I have read and am aware of the information provided, and have the opportunity to raise questions. I voluntarily participate in the survey and can withdraw at any time without reason. If you agree and acknowledge the above content, please click the "Agree" button below to begin answering the questions. If you do not agree or acknowledge the content, please click the "Disagree" button below to interrupt this survey project.

If you agree to participate in our survey, please click 'Agree' below and start the questionnaire survey Single Choice Question*

| ○ Agree |
| --- |
| ○ Disagree(Please skip to the end of the questionnaire and submit your answer sheet) |

**1、 General Information [16 Questions]**

1.1 Your name: [fill in the blank question]*

_________________________________

1.2 Your location[Single Choice Question]*

| ○ New urban area |
| --- |
| ○ Saihan District |
| ○ Hui Muslim District |
| ○ Yuquan District |
| ○ Tu Zuoqi |
| ○ Wuchuan County |
| ○ Qingshuihe County |
| ○ Lin County |
| ○ Tuo County |

1.3 Your phone number: [fill in the blank question]*

_________________________________

1.4 What is your year of birth: [Single Choice Question]*

| ○1964 |
| --- |
| ○1965 |
| ○1966 |
| ○1967 |
| ○1968 |
| ○1969 |
| ○1970 |
| ○1971 |
| ○1972 |
| ○1973 |
| ○1974 |
| ○1975 |
| ○1976 |
| ○1977 |
| ○1978 |
| ○1979 |
| ○1980 |
| ○1981 |
| ○1982 |
| ○1983 |
| ○1984 |
| ○1985 |
| ○1986 |
| ○1987 |
| ○1988 |
| ○1989 |
| ○1990 |
| ○1991 |
| ○1992 |
| ○1993 |
| ○1994 |
| ○1995 |
| ○1996 |
| ○1997 |
| ○1998 |
| ○1999 |
| ○2000 |

1.5 Your highest education level (highest degree obtained): [Single choice question]*

| ○ No formal education received |
| --- |
| ○Elementary school |
| ○ Junior high school |
| ○ Vocational school |
| ○ High School |
| ○ Junior college |
| ○ Undergraduate degree |
| ○ Master's students |
| ○ Doctoral students |

1.6 Current or most recent occupation (prior to retirement if applicable) [Single choice question]*

| ○ Heads of state organs, party mass organizations, enterprises and institutions |
| --- |
| ○ Professional technical personnel (such as accountants, lawyers, architects, journalists, etc., excluding medical personnel) |
| ○ Clerks and related personnel |
| ○ Business and service industry personnel |
| ○ Production personnel in agriculture, forestry, animal husbandry, fishing, and water conservancy industries |
| ○ Production and transportation equipment operators and related personnel |
| ○ Soldiers |
| ○ Other practitioners |

1.7 What is your household’s average monthly income per person? (in yuan) [Single choice question]*

| ○≤1000 |
| --- |
| ○1001-2000 |
| ○2001-3000 |
| ○3001-4000 |
| ○4001-5000 |
| ○5001-6000 |
| ○6001-8000 |
| ○8001-10000 |
| ○≥10001 |

1.8 You **currently** how to bear medical expenses: [Multiple choice question] *

| □ Publicly funded medical care |
| --- |
| □ Resident medical insurance |
| □ Employee medical insurance |
| □ New Rural Cooperative Medical Scheme |
| □ Commercial medical insurance |
| □ Uninsured (out-of-pocket payments) |

1.9 What is your marital status? [Single Choice Question]*

| ○ Unmarried (single) |
| --- |
| ○ Married |
| ○ Divorce |
| ○ Widowed |
| ○ Unmarried (not single) |

1.10 How many times have you given birth in the past? [Single Choice Question]*

| ○0 |
| --- |
| ○1 |
| ○2 |
| ○3 |
| ○≥4 |

1.11 Do you have a history of reproductive tract diseases? [Single Choice Question]*

| ○ Yes |
| --- |
| ○ None |

1.12 Have any first-degree relatives (parents, siblings, children) been diagnosed with cancer? [Single Choice Question]*

| ○ Yes |
| --- |
| ○ None |

1.13 How is your smoking status [Single Choice Question]*

| ○Never smoked before |
| --- |
| ○ Have a history of smoking, but have quit smoking |
| ○Still smoking |

1.14 How is your drinking situation [Single Choice Question]*

| ○I have never consumed alcohol before |
| --- |
| ○ Have a history of drinking alcohol, but have quit drinking |
| ○Still drinking |

**2、 Knowledge related to cervical cancer** (The following is only a health knowledge survey and not a test. Please choose the answer you think is correct. If you are not sure about the correct answer, please select "Unsure". This section is a single choice.)

2.1 Have you heard of cervical cancer? [Single Choice Question]*

| ○ Yes |
| --- |
| ○ No(Please skip to question 31) |

2.2 Is human papillomavirus (HPV) infection the main cause of cervical cancer? [Single Choice Question]*

| ○ Yes |
| --- |
| ○ No |
| ○Unsure |

2.3 Is HPV virus transmitted through sexual activity? [Single Choice Question]*

| ○ Yes |
| --- |
| ○ No |
| ○Unsure |

2.4 Does premature sexual activity increase the risk of cervical cancer? [Single Choice Question]*

| ○ Yes |
| --- |
| ○ No |
| ○Unsure |

2.5 Does having multiple sexual partners increase the risk of cervical cancer? [Single Choice Question]*

| ○ Yes |
| --- |
| ○ No |
| ○Unsure |

2.6 Does early childbirth (before the age of 18) increase the risk of cervical cancer? [Single Choice Question]*

| ○ Yes |
| --- |
| ○ No |
| ○Unsure |

2.7 Can only women be infected with HPV virus? [Single Choice Question]*

| ○ Yes |
| --- |
| ○ No |
| ○Unsure |

2.8 Does consistent condom use reduce HPV transmission risk? [Single Choice Question]*

| ○ Yes |
| --- |
| ○ No |
| ○Unsure |

2.9 Can early cervical cancer be cured? [Single Choice Question]*

| ○ Yes |
| --- |
| ○ No |
| ○Unsure |

2.10 Can cervical cancer be detected early through cervical cancer screening? [Single Choice Question]*

| ○ Yes |
| --- |
| ○ No |
| ○Unsure |

2.11 Can rural women aged 11.35-64 undergo free cervical cancer screening? [Single Choice Question]*

| ○ Yes |
| --- |
| ○ No |
| ○Unsure |

2.12 Is HPV vaccination effective in preventing cervical cancer? [Single Choice Question]*

| ○ Yes |
| --- |
| ○ No |
| ○Unsure |

2.13 Which populations are recommended for HPV vaccination? [Multiple Choice Question] *

| □ Male only |
| --- |
| □ Female only |
| □ Both men and women need to be vaccinated |
| □ Unsure |

2.14 What is the best time to receive the HPV vaccine? [Single Choice Question]*

| ○ Before the first sexual encounter |
| --- |
| ○ After the first sexual encounter |
| ○ You can do it anytime |
| ○ Unsure |

2.15 Do I still need to undergo cervical cancer screening after receiving the HPV vaccine? [Single Choice Question]*

| ○ Yes |
| --- |
| ○ No |
| ○ Unsure |

**3、 Attitude towards cervical cancer screening and HPV vaccination** ( Do you agree with the following views/statements? Each item needs to be answered. Please choose the option that is closest to your viewpoint.)

3.1 As a female, everyone is likely to have cervical cancer, including myself. [Single choice question]*

| ○ Strongly agree |
| --- |
| ○ Agree |
| ○ Can't explain clearly |
| ○ Disagree |
| ○ Strongly disagree |

3.2 Everyone may be infected with HPV virus, including myself. [Single choice question]*

| ○ Strongly agree |
| --- |
| ○ Agree |
| ○ Can't explain clearly |
| ○ Disagree |
| ○ Strongly disagree |

3.3 Cervical cancer is a serious disease. [Single choice question]*

| ○ Strongly agree |
| --- |
| ○ Agree |
| ○ Can't explain clearly |
| ○ Disagree |
| ○ Strongly disagree |

3.4 Regular cervical cancer screening can make me feel at ease about my health. [Single choice question]*

| ○ Strongly agree |
| --- |
| ○ Agree |
| ○ Can't explain clearly |
| ○ Disagree |
| ○ Strongly disagree |

3.5 I think cervical cancer screening is a manifestation of family responsibility. [Single choice question]*

| ○ Strongly agree |
| --- |
| ○ Agree |
| ○ Can't explain clearly |
| ○ Disagree |
| ○ Strongly disagree |

3.6 Cervical cancer screening needs to contact private parts, which will make me feel embarrassed/embarrassed. [Single choice question]*

| ○ Strongly agree |
| --- |
| ○ Agree |
| ○ Can't explain clearly |
| ○ Disagree |
| ○ Strongly disagree |

3.7 If the process of cervical cancer screening is painful, I will not want to go to the screening. [Single choice question]*

| ○ Strongly agree |
| --- |
| ○ Agree |
| ○ Can't explain clearly |
| ○ Disagree |
| ○ Strongly disagree |

3.8 I think participating in cervical cancer screening is a waste of my time. [Single choice question]*

| ○ Strongly agree |
| --- |
| ○ Agree |
| ○ Can't explain clearly |
| ○ Disagree |
| ○ Strongly disagree |

3.9 I think HPV vaccination can prevent HPV infection. [Single choice question]*

| ○ Strongly agree |
| --- |
| ○ Agree |
| ○ Can't explain clearly |
| ○ Disagree |
| ○ Strongly disagree |

3.10 I think HPV vaccination can prevent cervical cancer. [Single choice question]*

| ○ Strongly agree |
| --- |
| ○ Agree |
| ○ Can't explain clearly |
| ○ Disagree |
| ○ Strongly disagree |

3.12 I am worried that there is no effect on disease prevention after HPV vaccination. [Single choice question]*

| ○ Strongly agree |
| --- |
| ○ Agree |
| ○ Can't explain clearly |
| ○ Disagree |
| ○ Strongly disagree |

3.13 I am worried about serious adverse reactions caused by HPV vaccination. [Single choice question]*

| ○ Strongly agree |
| --- |
| ○ Agree |
| ○ Can't explain clearly |
| ○ Disagree |
| ○ Strongly disagree |

3.14 If it is difficult to make an appointment for HPV vaccine, I will not want to be vaccinated. [Single choice question]*

| ○ Strongly agree |
| --- |
| ○ Agree |
| ○ Can't explain clearly |
| ○ Disagree |
| ○ Strongly disagree |

3.15 If the price of HPV vaccine is very expensive, I will not want to be vaccinated. [Single choice question]*

| ○ Strongly agree |
| --- |
| ○ Agree |
| ○ Can't explain clearly |
| ○ Disagree |
| ○ Strongly disagree |

3.16 If my family or friends around me suffer from cervical cancer, it will increase my willingness to participate in cervical cancer screening and vaccination. [Single choice question]*

| ○ Strongly agree |
| --- |
| ○ Agree |
| ○ Can't explain clearly |
| ○ Disagree |
| ○ Strongly disagree |

3.17 People who have some knowledge of cervical cancer screening and HPV vaccine will be more willing to participate in screening and vaccination. [Single choice question]*

| ○ Strongly agree |
| --- |
| ○ Agree |
| ○ Can't explain clearly |
| ○ Disagree |
| ○ Strongly disagree |

3.18 People around me are participating in cervical cancer screening and vaccination, which will increase my willingness to screen and vaccinate. [Single choice question]*

| ○ Strongly agree |
| --- |
| ○ Agree |
| ○ Can't explain clearly |
| ○ Disagree |
| ○ Strongly disagree |

3.19 The doctor's recommendation of cervical cancer screening and HPV vaccine will increase my willingness to screen and vaccinate. [Single choice question]*

| ○ Strongly agree |
| --- |
| ○ Agree |
| ○ Can't explain clearly |
| ○ Disagree |
| ○ Strongly disagree |

**4、 Please choose the option that suits your actual situation. There is no right or wrong between the options, and we will not disclose your personal privacy**

**4.1 In the past two weeks** do you often encounter the following problems? [Matrix Single choice question]*

|  | Never | Occasionally (1-3 days) | Frequently (4-10 days) | Almost daily（11-14 days） |
| --- | --- | --- | --- | --- |
| Feeling dull or unwilling to do anything | ○ | ○ | ○ | ○ |
| Low mood, depression, or despair | ○ | ○ | ○ | ○ |
| Difficulty falling asleep; Waking up in the middle of the night, or conversely, having too much sleep time | ○ | ○ | ○ | ○ |
| Feeling tired or lacking vitality | ○ | ○ | ○ | ○ |
| Poor appetite or overeating | ○ | ○ | ○ | ○ |
| Disliking oneself - feeling that one is not doing well, disappointed in oneself, or has disappointed family expectations | ○ | ○ | ○ | ○ |
| Difficulty concentrating on tasks, such as reading newspapers or watching TV | ○ | ○ | ○ | ○ |
| Others have reported that you are slow to move or speak; Or conversely, you are more active than usual - restless and unable to stop | ○ | ○ | ○ | ○ |
| Recurrent thoughts of death or self-injury | ○ | ○ | ○ | ○ |

4.2 According to **past two weeks** regarding the situation, please answer whether there are any of the following described situations and frequencies. Please choose the most suitable answer for you to the following questions. [Matrix Single choice question]*

|  | Never | Occasionally (1-3 days) | Frequently (4-10 days) | Almost daily（11-14 days） |
| --- | --- | --- | --- | --- |
| Feeling nervous, anxious, or eager | ○ | ○ | ○ | ○ |
| It's hard to relax | ○ | ○ | ○ | ○ |
| Feeling scared that something terrible is about to happen | ○ | ○ | ○ | ○ |

4.3 Please carefully recall **within the past month** what is the frequency of the following symptoms in your life? [Matrix Single choice question]*

|  | Not satisfied | Dissatisfied | Neutral | Satisfied | Very satisfied |
| --- | --- | --- | --- | --- | --- |
| Due to unforeseen circumstances, I often feel restless and upset | ○ | ○ | ○ | ○ | ○ |
| I am confident in my ability to solve my personal problems | ○ | ○ | ○ | ○ | ○ |
| I feel that everything is going smoothly and as planned | ○ | ○ | ○ | ○ | ○ |
| Difficult things pile up like mountains, which one cannot overcome on their own | ○ | ○ | ○ | ○ | ○ |

4.4 Please choose the most suitable option for you [Matrix Single choice question]*

|  | Strongly disagree | disagree | Neutral | agree | Strongly agree |
| --- | --- | --- | --- | --- | --- |
| When faced with difficult tasks, I am confident that I can complete them | ○ | ○ | ○ | ○ | ○ |
| I will be able to successfully overcome many challenges | ○ | ○ | ○ | ○ | ○ |
| I am confident that I can effectively complete many different tasks | ○ | ○ | ○ | ○ | ○ |

4.5 Please indicate to what extent you agree or disagree with the following statements. [Matrix Single choice question]*

|  | Strongly disagree | Slightly disagree | Neutral | Slightly agree | Strongly agree |
| --- | --- | --- | --- | --- | --- |
| In my family, we support each other | ○ | ○ | ○ | ○ | ○ |
| In my family, I feel secure in family relationships | ○ | ○ | ○ | ○ | ○ |
| In my family, we help each other seek medical services (such as registration) when needed | ○ | ○ | ○ | ○ | ○ |
| In my family, we help each other make changes for the sake of health | ○ | ○ | ○ | ○ | ○ |
| In my family, even in difficult times, we maintain hope | ○ | ○ | ○ | ○ | ○ |
| In my family, we don't trust medical staff | ○ | ○ | ○ | ○ | ○ |
| When we encounter problems in school or work, our family can seek help from people outside of our family | ○ | ○ | ○ | ○ | ○ |
| If we need financial assistance, our family can borrow money from people outside of our family (such as 1000 yuan) | ○ | ○ | ○ | ○ | ○ |
| In the past 12 months, after covering basic living expenses, our family has no spare money left | ○ | ○ | ○ | ○ | ○ |
| In the past 12 months, my family's housing has not been able to meet their needs | ○ | ○ | ○ | ○ | ○ |

4.6 From very easy to very difficult, please evaluate the difficulty level of the following behaviors for you.

[Matrix Single choice question]*

|  | Very easy. | easily | difficulty | very difficult |
| --- | --- | --- | --- | --- |
| Find treatment information for your illness | ○ | ○ | ○ | ○ |
| Assessing the advantages and disadvantages of different treatment options | ○ | ○ | ○ | ○ |
| Find information on how to deal with mental health issues such as stress or depression | ○ | ○ | ○ | ○ |
| Determine the type of vaccine you may need to receive | ○ | ○ | ○ | ○ |

4.7 There are a total of 3 questions below, each of which is used to describe whether you are appropriate or to what extent the content of each question corresponds to you Please choose the most suitable answer for each of the following questions.[Matrix Single choice question]*

|  | Strongly disagree | disagree | Slightly disagree | Neutral | Slightly agree | agree | Strongly agree |
| --- | --- | --- | --- | --- | --- | --- | --- |
| I can get emotional help and support from my family when I need it | ○ | ○ | ○ | ○ | ○ | ○ | ○ |
| My friends really helped me | ○ | ○ | ○ | ○ | ○ | ○ | ○ |
| When I encounter problems, some people (relatives, neighbors, colleagues) will appear beside me | ○ | ○ | ○ | ○ | ○ | ○ | ○ |

4.8 To what extent can these sentences describe your personality? There is no right or wrong answer.

[Matrix Single choice question]*

|  | 1=Completely inconsistent | 2=Most of them do not meet the requirements | 3=somewhat inconsistent | 4=somewhat consistent | 5=Most of them comply | 6=fully compliant |
| --- | --- | --- | --- | --- | --- | --- |
| I often feel scared | ○ | ○ | ○ | ○ | ○ | ○ |
| Once the goal is set, I will persist in striving to achieve it | ○ | ○ | ○ | ○ | ○ | ○ |
| I think most people are basically kind-hearted | ○ | ○ | ○ | ○ | ○ | ○ |
| My mind is often filled with vivid images | ○ | ○ | ○ | ○ | ○ | ○ |
| I find crowded gatherings boring | ○ | ○ | ○ | ○ | ○ | ○ |
